# A Polyphenol-Rich Extract from Muscadine Grapes Prevents Hypertension-Induced Diastolic Dysfunction and Oxidative Stress

**DOI:** 10.3390/antiox11102026

**Published:** 2022-10-14

**Authors:** Pooja D. Patil, Ana Clara Melo, Brian M. Westwood, E. Ann Tallant, Patricia E. Gallagher

**Affiliations:** Surgery/Hypertension and Vascular Research, Wake Forest University School of Medicine, Winston-Salem, NC 27101, USA

**Keywords:** muscadine grape, polyphenols, hypertension, cardiac damage, oxidative stress, superoxide dismutase, catalase, 8-hydroxy-2′-deoxyguanosine

## Abstract

Muscadine grapes are abundant in dietary polyphenols, but their effect on hypertension-induced cardiac damage is limited. This study assessed whether a muscadine grape skin/seed extract supplement (MGES) prevents hypertension-induced cardiac damage and oxidative stress. Male Sprague Dawley rats were treated for four weeks with drinking water, angiotensin II (Ang II) to induce hypertension, MGES, or both Ang II and MGES. Cardiac function assessed by echocardiography showed that Ang II increased systolic blood pressure while MGES alone or in combination with Ang II had no effect. Ang II increased E/e′, an indicator of left ventricular filling pressure and diastolic dysfunction, by 41% compared to Control and co-treatment with MGES prevented the Ang II-mediated increase, suggesting that the extract attenuated hypertension-induced diastolic function. Ang II infusion increased urinary 8-hydroxy-2′-deoxyguanosine and cardiac 4-hydroxynonenal and malondialdehyde, which were prevented by the extract. The antioxidant enzymes catalase and superoxide dismutase 1 activity and mRNA were increased significantly in animals treated with MGES alone or in combination with Ang II, suggesting that the extract upregulates oxidative stress defense mechanisms in cardiac tissue. Thus, MGES may serve as a medical food to protect the heart from hypertension-induced diastolic dysfunction caused in part by excessive reactive oxygen species production.

## 1. Introduction

Hypertension currently afflicts over a billion adults worldwide, but less than half of this population have their blood pressure under control [1]. Uncontrolled hypertension can lead to pathological cardiac hypertrophy, systolic and diastolic abnormalities, and eventually, heart failure. During early stages of hypertensive cardiac remodeling, the heart undergoes compensatory cardiac hypertrophy to preserve function. However, the increase in afterload also results in reduced compliance of the cardiac muscle [2]. Ultimately, diastolic and systolic abnormalities occur and can lead to congestive heart failure. 

Angiotensin II (Ang II) is implicated in the pathogenesis of adverse hypertensive cardiac remodeling, especially in the progression of hypertrophy and fibrosis. The pathological effects of Ang II on the heart are mediated through the angiotensin receptor subtype 1 or AT_1_ receptor [3]. Both angiotensin converting enzyme inhibitors (ACEIs), which prevent the production of Ang II from Ang I, and Ang II receptor blockers (ARBs), which inhibit hormone binding to the AT_1_ receptor, are effective in treating hypertension as well as the accompanying cardiac fibrosis and hypertrophy. However, ACEIs and ARBs can cause adverse side effects, including cough, hypotension, and angioedema [4]. 

Although ACEIs, ARBs, diuretics, β-blockers, and calcium channel blockers are effective pharmaceuticals used for the treatment of hypertension, one of the first-line interventions for cardiovascular disease is improving dietary health [5]. A primary focus of dietary intervention is to reduce the ingestion of highly processed foods, replacing them with fruits and vegetables that are high in polyphenols and associated with a decrease in cardiovascular risk [6]. In particular, the high contents of dietary polyphenols in grapes, tea, and cocoa have antioxidant properties that improve cardiovascular risk factors. A meta-analysis of the effects of red table grape seed extracts on cardiovascular health demonstrated a modest improvement in systolic blood pressure and heart rate in patients with moderate or high risk of cardiovascular disease [7]. Green tea improves cardiovascular outcomes [8] and all-cause mortality, in part due to its high phenolic content [9]. Epigallocatechin-3 gallate (EGCG), a polyphenol predominantly found in green tea, ameliorates pressure overload-induced cardiac hypertrophy, fibrosis and systolic function in rodents [10]. These results suggest that tea and grape polyphenols improve cardiovascular health.

Muscadine grapes (*Vitis rotundifolia*) have a high antioxidant capacity and a phenolic profile that is distinct from European grapes. The phenolic content is highest in muscadine grape skins and seeds [11]. Some of the major compounds identified in muscadine grapes include tannins, anthocyanins, flavan-3-ols, flavonoids, and ellagic acid derivatives; in contrast, the resveratrol content is relatively low. While once considered a waste product, ground muscadine skin and seeds are now sold as a nutritional supplement. Clinical trials in cancer patients showed beneficial outcomes following administration of commercial muscadine grape supplements [12,13,14]. The aim of this study was to investigate the potential of a commercial polyphenol-enriched muscadine grape and skin extract supplement (MGES) to prevent Ang II-mediated hypertension and hypertension-associated cardiac damage.

## 2. Materials and Methods

### 2.1. Animal Model

The Wake Forest School of Medicine Animal Care and Use Committee approved all animal experiments. The care and use of animals were in accordance with all applicable international, national, and institutional standards. The animals were administered in accordance with the ethical guidelines established at Wake Forest University School of Medicine. The authors complied with the ARRIVE guidelines [15].

Sprague Dawley rats (male, 6 weeks of age, *n* = 8/treatment group) had ad libitum access to standard rat chow (5P00, LabDiet, Richmond, IN; 22% crude protein, 5% crude fat, 5% crude fiber and 6% ash). Rats were randomized prior to any treatment. Rats in the MGES or Ang II/MGES groups received MGES at 7 weeks of age so that the MGES was onboard prior to the treatment with Ang II. At 8 weeks of age, Ang II treatment was initiated (in the Ang II and Ang II/MGES group) and the MGES was continued in the MGES and Ang II/MGES groups for an additional 4 weeks of treatment for all groups. The treatments for each group were regular drinking water (Control), MGES (0.2 mg/mL of phenolics in their drinking water), human Ang II (Bachem, Switzerland; 24 µg/kg/h via implanted osmotic mini-pump), or both Ang II and MGES. Rats were sedated with isoflurane before subcutaneous implantation of an osmotic mini-pump (Alzet model 2004) into the back that delivered Ang II continuously for the four weeks of treatment. During the week of sacrifice (week 4 of treatment), rats were placed in metabolic cages for the measurement of food and water intake and for a 24 h urine collection.

### 2.2. Muscadine Grape Extract

The MGE supplement used in this study is a dried water extract of MG skin and seed purchased from Piedmont Research Development Corporation (Advance, North Carolina, USA). This supplement is distinct from other commercially available MGE supplements that are composed of ground skins and/or seeds and are not extracted. A previous study reported that the major components in the extract were epicatechin, gallic acid, procyanidin B, ellagic acid, catechin, and catechin gallate; resveratrol was not detected [16]. For administration to animals, the powder was resuspended in deionized water, stirred for 2 h and filtered through Whatman #4 filter paper under vacuum, resulting in a clarified aqueous MGES, which was provided to the rats in their drinking water. Control rats received deionized water. The liquid extract was prepared twice weekly and the water bottles for the rats were changed at least twice weekly. The MGES preparation was standardized to total phenolics/mL water, measured using the Folin-Ciocalteau reagent with gallic acid used as a standard. Gallic acid was diluted in 70% ethanol and a concentration curve of 12.5, 25, 50 and 75 mg/mL was prepared. The Folin–Ciocalteu reagent (0.5 mL of a 1:10 dilution) was added to 100 μL of the gallic acid standards or the experimental samples and the mixtures were incubated for 1–8 min at room temperature followed by the addition of 400 μL of 7.5% sodium carbonate. After a 30 min incubation at 40 °C, the absorbance was read at 760 nm. The phenolic concentration of each resuspended preparation was tested and varied little over the time course of the experiment (0.42 ± 0.002 mg/mL, *n* = 428 preparations).

### 2.3. Blood Pressure and Echocardiography

Conscious rats were trained at three separate training sessions on a non-invasive blood pressure monitor (Columbus Instruments, Columbus, OH, USA), at six weeks of age. Systolic blood pressure was recorded weekly via tail-cuff plethysmography starting at seven weeks of age (baseline), after MGES pretreatment at 8 weeks of age (week 0) and during each week of treatment (weeks 1–4). Blood pressure was measured for each animal, at the same time of day by the same individual, and reported as the mean of the three measurements. Echocardiography was performed using a small animal ultrasound Vevo 2100 High-Resolution Imaging System (VisualSonics Fujifilm, Toronto, ON, Canada) with an MS250 (13–24 MHz) transducer. Rats were mildly sedated with 1–2% isoflurane. Scans of the short axis M-mode (at mid-papillary level) were used to quantify systolic function, left ventricular posterior wall thickness (LVPWT), end diastolic diameter (EDD), and posterior wall thickness (PWT). Scans to estimate diastolic function were taken in Pulse Wave Doppler mode in 4-chamber view to quantify isovolumic relaxation time (IVRT) and E (peak velocity of blood flow during early diastole) and in Tissue Doppler mode in 4-chamber view at the level of the mitral annulus to measure e′ (peak mitral annular velocity during early diastole).

### 2.4. Immunohistochemistry

Five-micron sections of paraffin-embedded hearts were stained with wheat germ agglutinin (Alexa Fluor^TM^; 488 Conjugate, #W11261, Invitrogen, Carlsbad, CA, USA), or target-specific antibodies. The Opal^TM^ Multiplex IHC kit (Perkin Elmer, Waltham, MA, USA) was used to stain with various antibodies; antigen retrieval was performed using either Antigen Retrieval pH6 buffer (AR6; PerkinElmer) or Tris-EDTA pH9 buffer (TE9), and non-specific binding was inhibited using serum-free protein block (Dako, Agilent, Santa Clara, CA, USA). The antibodies used were purchased from Abcam (Cambridge, UK) and include: 4-HNE (4-hydroxynonenol Ab46545; 1:250; TE9), MDA (malondialdehyde Ab6463; 1:100; TE9), SOD1 (superoxide dismutase 1 Ab13498;1:100; TE9), catalase (Ab16731; 1:100; TE9) and secondary antibody (anti-rabbit HRP IgG, #NEF812E001EA, Perkin Elmer, Waltham, MA, USA). Slides were incubated with primary antibodies overnight at 4 °C, washed 3 times in PBS and incubated with secondary antibody for 1 h at room temperature. Slides were subsequently incubated with Opal^TM^ TSA fluorophores (Perkin Elmer) diluted in 1x Plus amplification Buffer (Perkin Elmer Waltham, MA, USA; FP1498) at room temperature for 10 min in low light to label antibody bound to the tissue. Second heat-mediated antigen retrieval in AR6 buffer was performed to adhere the fluorophore to the tissue and remove the antibody complex. DAPI (Spectral DAPI, Perkin Elmer) was used as a nuclear counterstain. Tissues were mounted with a coverslip using Prolong Antifade Mountant (Thermo Fisher, Waltham, MA, USA) and dried overnight in the dark. Images from four representative regions of each heart section were acquired with a Mantra microscope (Perkin Elmer) at 400× magnification. Mean cross-sectional area of the cardiomyocytes in tissues stained with wheat germ agglutinin was obtained using inForm software (Perkin Elmer).

### 2.5. Analysis of Immunohistochemistry

Slides with fluorophore-labelled secondary antibodies were processed using inForm software. A slide without immunofluorescence tags was used as a control for tissue autofluorescence. The specific tissue fluorescence less the background autofluorescence was quantified as the optical density (OD). The upper inner fence (UIF) was calculated from the quantified OD obtained from all images across the groups and used as the cut-off for positive staining. The UIF provides a standardized method to identify outliers [17]. For images stained with WGA to quantify the mean cross-sectional area of cardiomyocytes, inForm identified cardiomyocytes by creating a “shell” that extended from a positive DAPI signal to the positive staining of the WGA. Any measurements outside of 2.5 standard deviations from the mean in each group were eliminated.

### 2.6. Quantification of 8-Hydroxy-2′-deoxyguanosine (8-OHdG)

A 24 h urine sample was collected using metabolic cages the day before the rats were sacrificed. Urinary 8-OHdG was measured using an in vitro ELISA kit (ab201734, Abcam, Cambridge, UK). Urinary 8-OHdG data are expressed as ng/mL/24 h.

### 2.7. Quantification of Catalase and Superoxide Dismutase Activities

Cardiac tissue from the left ventricle was homogenized in 10 mM Tris-hydrochloric acid (Tris-HCl; pH 7.4) at 4 °C and the homogenate was subjected to centrifugation at 4000× *g* for 30 min at 4 °C. The resulting supernatant was collected, and protein concentrations were determined using the protein Bio-Rad assay [18]. The activities of endogenous cardiac catalase and superoxide dismutase were measured using the enzymatic assay kit from Cayman Chemical (Ann Arbor, MI, USA) according to the manufacturer’s protocol.

### 2.8. RNA Isolation/qRT-PCR

RNA, isolated from the left ventricle of each rat heart using the TRIzol reagent (Thermo Fischer Scientific, Waltham, MA) was measured by RT-PCR, as previously described [19]. The primer sets for catalase (Zm04059155_m1, catalog number 4351372) and SOD1 (Hs00533490_m1, catalog number 4331182) were purchased from ThermoFisher Scientific. 18S ribosomal RNA was used as a control and the results were quantified as the C_t_ values, where C_t_ is defined as the threshold cycle of PCR at which the amplified product is first detected; the values are expressed as the ratio of target to control using the equation 2^-ΔΔCt^ and referred to as Relative Gene Expression. The value of the Control was set at 1, and all data were calculated as fold change compared to Control.

### 2.9. Statistics

Data are presented as mean ± standard error of the mean (SEM). Blood pressure was analyzed by Repeated Measures ANOVA with post-test comparisons to baseline using Dunnett’s test. Correlations between two variables were tested by linear regression. All other data were subjected to one-way analysis of variance (ANOVA) with post-test analysis by Tukey’s multiple comparisons tests, using GraphPad Prism 6.0 (http://www.graphpad.com, GraphPad Software Inc., La Jolla, CA, USA). Sample size was chosen to provide a sufficient number of animals to detect a 1.5 standard deviation difference between groups with 80% power at a 5% two-sided level of significance. The criterion for statistical significance was *p* < 0.05.

## 3. Results

### 3.1. Blood Pressure and Cardiac Morphology

**Figure 1:**
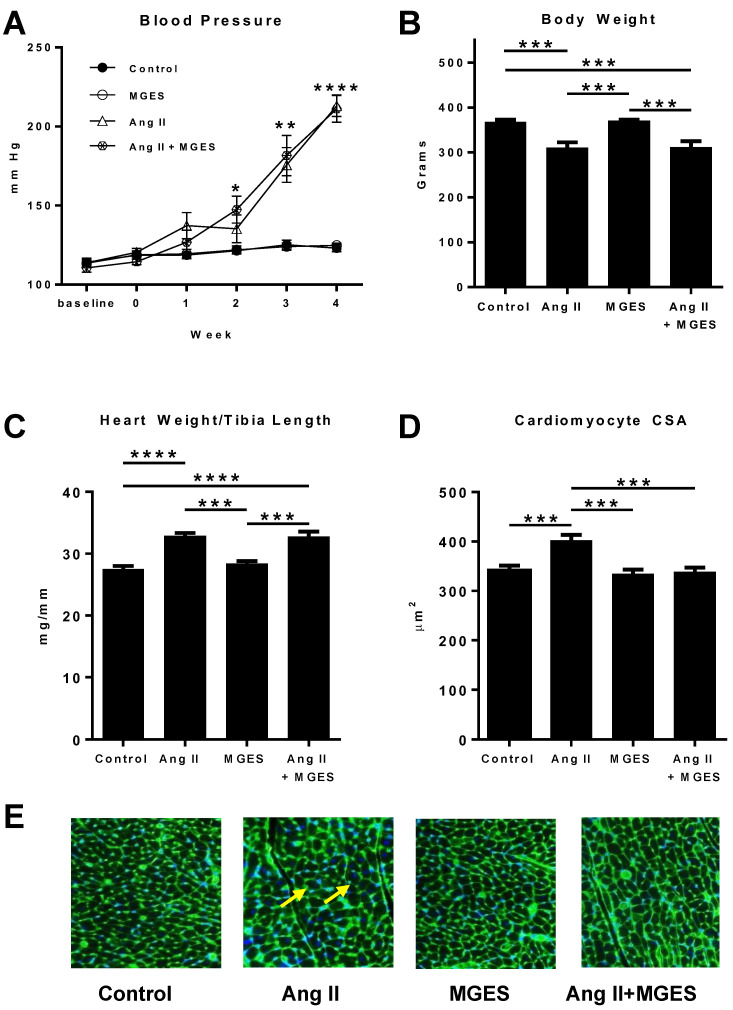
The effect of MGES on Ang II-induced changes in blood pressure, body weight, and cardiac hypertrophy. (**A**) Blood pressure at baseline, prior to MGES treatment (week 0) and for the 4 weeks of treatment, (**B**) body weight, (**C**) heart weight/tibia length ratio at the time of sacrifice, and (**D**,**E**) mean cross-sectional area of the left ventricles measured in rats administered drinking water (Control), Ang II alone (Ang II), MGES alone (MGES), or a combination of MGES and Ang II (Ang II + MGES). Hypertrophic myocytes are indicated by the yellow arrows. Values are mean ± SEM; *n* = 8/group. The asterisk (*) represents statistically significant comparisons. * *p* < 0.05; ** *p* < 0.01; *** *p* < 0.001; **** *p* < 0.0001.

Systolic blood pressure was measured in male Sprague Dawley rats treated with Ang II, in the presence or absence of MGES. Basal blood pressure, either before (baseline) or after the one-week pretreatment with MGES (week 0), was not significantly different across groups (mean systolic blood pressure at baseline: 113 ± 1.1 mm Hg, mean systolic blood pressure after pre-treatment with MGES at week 0: 118 ± 1.2 mm Hg). After 4 weeks, Ang II treatment resulted in a time-dependent 73% increase in systolic blood pressure compared to Control after 4 weeks (*p* < 0.0001, Figure 1A). MGE supplementation in either normotensive or hypertensive rats had no effect on blood pressure. Ang II reduced body weight by 16% as compared to Control (*p* < 0.001); MGES did not prevent the reduction in body weight (Figure 1B). Food consumption, water intake, and urine volume were measured prior to euthanasia, by placing the rats in metabolic cages for 24 h. Food consumption, measured as a function of the body weight of the rats, increased with Ang II treatment but was not affected by MGES in the presence or absence of Ang II. Food consumption was greater in Ang II-treated rats (76.1 ± 5.6 g/kg) or Ang II/MGES-treated rats (81.2 ± 3.9 g/kg) compared with Control rats (57.1 ± 2.7 g/kg; *p* < 0.05 for Ang II and *p* < 0.01 for Ang II/MGES). No difference in food consumption was observed with MGES-treated rats (55.1 ± 2.9 g/kg) as compared to control rodents. Water intake and urine volume were increased by Ang II and not changed by MGES.

Heart weight-to-tibia length was used as a measure of gross cardiac hypertrophy. MGES had no effect on the increase in heart weight-to-tibia length induced by Ang II (Figure 1C), suggesting that the extract did not alter Ang II-induced gross cardiac hypertrophy. Importantly, ingestion of MGES alone did not induce cardiac hypertrophy. Ang II also increased the cross-sectional area of cardiac myocytes as compared to heart sections from control or MGES-treated rats (*p* < 0.001, Figure 1D). MGES alone did not affect myocyte size; however, the extract significantly blocked the Ang II-mediated increase in mean cross-sectional area of cardiomyocytes (*p* < 0.001 compared to Ang II alone). Staining of sections of the left ventricle with wheat germ agglutinin (green staining of the cell boundary, Figure 1E) showed that the size of the cardiomyocytes in the heart tissue from Ang II-treated rats was increased significantly compared to the tissue from the other groups.

### 3.2. Cardiac Function and 8-Hydroxy-2′-deoxyguanosine

Echocardiographic scans taken in parasternal short axis M mode were used to calculate systolic function and ventricular thickness. Neither ejection fraction (EF) nor fractional shortening (FS) was affected after 4 weeks of Ang II treatment, with or without MGES, (Table 1). In contrast, Ang II treatment or the associated increase in blood pressure significantly reduced the end diastolic internal diameter (EDD, *p* < 0.0001) and increased the left ventricular posterior wall thickness (LVPWT, *p* < 0.01), resulting in a 75% increase in relative wall thickness compared to Control (RWT, *p* < 0.0001). Co-treatment with MGES did not improve or exacerbate these morphometric parameters of cardiac hypertrophy. Echocardiographic scans were performed in pulsed wave Doppler mode to investigate mitral inflow and in tissue Doppler mode to quantify mitral annular movement. Ang II administration increased isovolumic relaxation time (IVRT), as shown in Table 1 (*p* < 0.0001); co-administration of MGES had no significant effect on IVRT.

**Figure 2:**
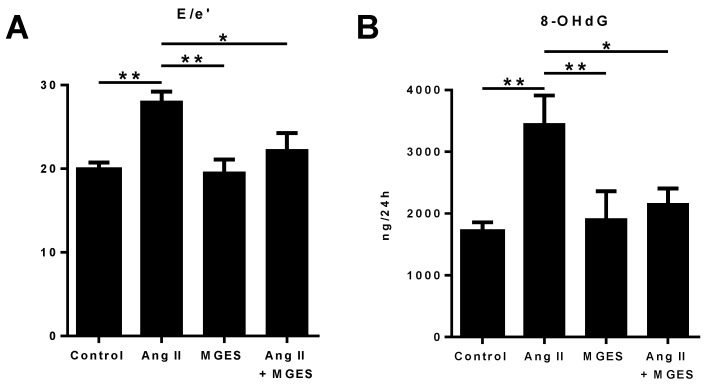
The effect of MGES on Ang II-mediated changes in diastolic function and 8-OHdG. (**A**) E/e′ was calculated from pulse wave Doppler and tissue Doppler mode in rats treated with either normal drinking water (Control), MGES and/or Ang II, as indicated, after 4 weeks of Ang II infusion. (**B**) Quantification of 8-OHdG in urine collected for 24 h after 4 weeks of treatment is shown for rats administered drinking water (Control), Ang II alone (Ang II), MGES alone (MGES), or a combination of MGES and Ang II (Ang II + MGES). Values are mean ± SEM; *n* = 8. The asterisk (*) represents statistically significant comparisons. * *p* < 0.05; ** *p* < 0.01.

E, peak velocity of blood flow in early diastole, and e′, peak velocity of the mitral annulus during early diastole, were used to evaluate diastolic function. Ang II increased E/e′, an indicator of left ventricular filling pressure, by 41% compared to Control (20.0 ± 0.80 vs. 28.1 ± 1.11, *p* < 0.01) and co-treatment with MGES prevented this increase as compared to Ang II alone (28.1 ± 1.1 vs. 22.3 ± 2.0, *p* < 0.05, Figure 2A), suggesting that the extract attenuates hypertension-induced cardiac dysfunction. A marker of oxidative stress, 8-hydroxy-2′-deoxyguanosine (8-OHdG), was quantified in a 24 h urine collection from each rat at the end of the treatment period. Ang II increased urinary 8-OHdG 2-fold (*p* < 0.05) as compared to 8-OHdG excretion in untreated rats (Figure 2B). Co-administration of MGES with Ang II infusion significantly reduced urinary 8-OHdG (*p* < 0.05 compared to Ang II alone); MGES alone had no effect, suggesting that the extract may prevent cardiac damage induced by hypertension through reduced oxidative stress.

### 3.3. Oxidative Stress

**Figure 3:**
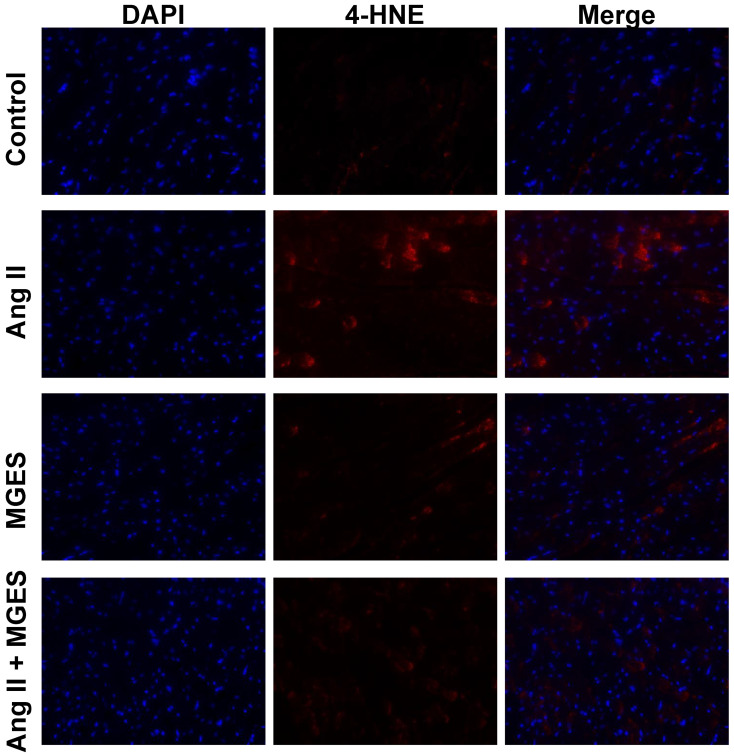
MGES prevented the Ang II-mediated increase in cardiac 4-HNE. Representative images of cardiac tissue stained with the nuclear counterstain DAPI (blue) and an antibody to 4-HNE (red) imaged at ×400 are shown for hearts from rats administered drinking water (Control), Ang II alone (Ang II), MGES alone (MGES), or a combination of MGES and Ang II (Ang II + MGES).

**Figure 4:**
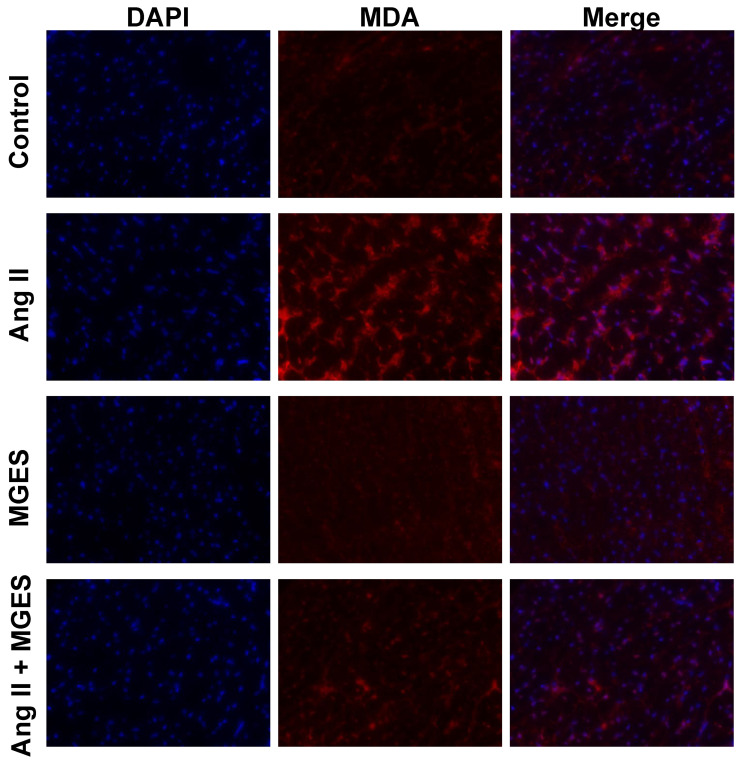
MGES prevented the Ang II-mediated increase in cardiac MDA. Representative images of cardiac tissue stained with the nuclear counterstain DAPI (blue) and an antibody to MDA (red) imaged at ×400 are shown for hearts from rats administered drinking water (Control), Ang II alone (Ang II), MGES alone (MGES), or a combination of MGES and Ang II (Ang II + MGES).

Oxidative stress plays a crucial role in the development of Ang II-induced cardiac damage. Therefore, markers of oxidative stress were assessed in the myocardium of rats treated with Ang II alone or co-treated with MGES. A significant increase in red fluorescent immunostaining for the lipid peroxidation marker 4-HNE (Figure 3) was observed in tissue sections from the hearts of rats treated with Ang II as compared to sections from control or MGES-treated rats. The merged images show the cellular 4-HNE immunofluorescence co-localized with the nucleus counterstained with DAPI. Co-administration of MGES with Ang II markedly reduced the immunostaining to control levels. Fluorescent immunostaining of cardiac MDA, a reactive aldehyde product of lipid peroxidation, was also increased in sections of rat heart following Ang II treatment as compared to Control, MGES, or Ang II + MGES (Figure 4). These results suggest that MGES protects the heart from oxidative damage caused by Ang II.

**Figure 5:**
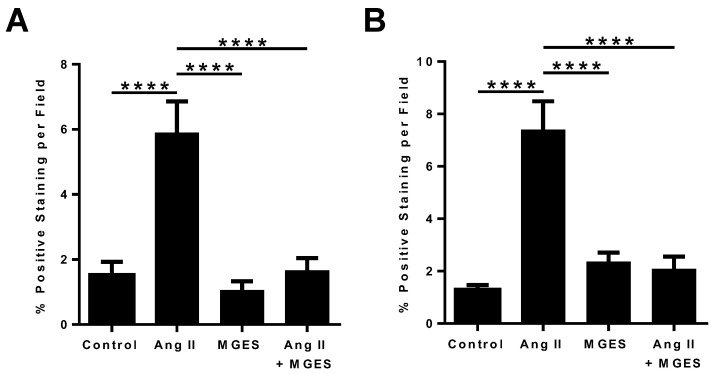
MGES ameliorated the Ang II-mediated increase in markers of oxidative stress. (**A**) Quantification of sections of cardiac tissue stained with an antibody to 4-HNE and (**B**) quantification of sections of cardiac tissue stained with an antibody to MDA are shown for hearts from rats administered drinking water (Control), Ang II alone (Ang II), MGES alone (MGES), or a combination of MGES and Ang II (Ang II + MGES). Four representative images per tissue were obtained using a Mantra microscope. Values are mean ± SEM; *n* = 8 per group. The asterisk (*) represents statistically significant comparisons. **** *p* < 0.0001.

Quantification of the immunohistochemical images showed that Ang II administration increased 4-HNE in cardiac tissue sections by 4-fold (*p* < 0.0001 compared to Control, Figure 5A). Co-administration of MGES prevented the Ang II-induced increase in 4-HNE (*p* < 0.0001 as compared to Ang II alone); MGE supplementation alone had no effect on 4-HNE. Cardiac MDA concentration was also higher in rat hearts following Ang II treatment (*p* < 0.0001 compared to Control, MGES, or Ang II + MGES; Figure 5B). MGES prevented the Ang II-induced increase in MDA (*p* < 0.0001 compared to Ang II alone, Figure 5B). The concentration of MDA following MGES treatment alone was not different than control.

### 3.4. Effect of MGES on Cardiac SOD1

**Figure 6:**
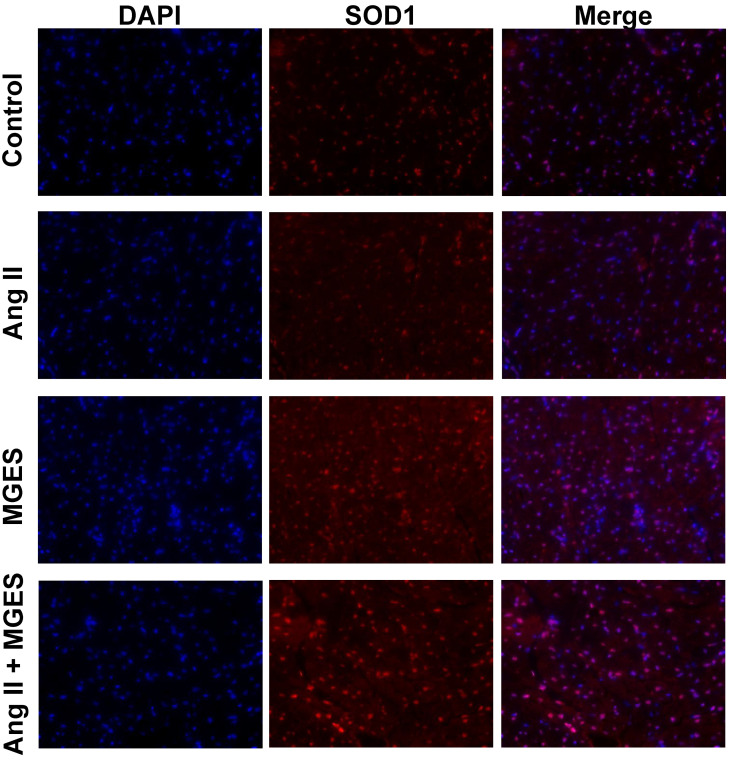
MGES increases SOD1 immunofluorescence in cardiac tissue. Representative images of cardiac tissue stained with the nuclear counterstain DAPI (blue) and an antibody to SOD1 (red) imaged at ×400 are shown for hearts from rats administered drinking water (Control), Ang II alone (Ang II), MGES alone (MGES), or a combination of MGES and Ang II (Ang II + MGES).

**Figure 7:**
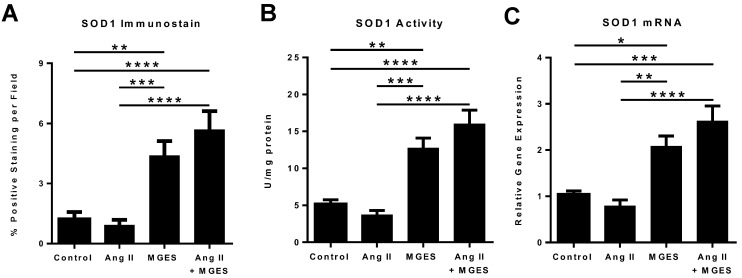
MGES increases SOD1 protein, activity and mRNA in normotensive and hypertensive rat hearts. SOD1 protein, activity or mRNA were measured in heart tissue from rats administered drinking water (Control), MGES alone (MGES), Ang II alone (Ang II) or a combination of MGES and Ang II (Ang II + MGES). (**A**) Quantification of cardiac tissue sections stained with an antibody to SOD1; (**B**) Quantification of SOD1 activity; (**C**) Quantification of SOD1 mRNA. Four representative images per tissue were obtained using the Mantra microscope. Values are mean ± SEM; *n* = 8 per group. The asterisk (*) represents statistically significant comparisons. * *p* < 0.05; ** *p* < 0.01; *** *p* < 0.001; **** *p* < 0.0001.

SOD1 is a powerful endogenous antioxidant enzyme that converts the superoxide radical into hydrogen peroxide. Immunohistochemical analysis of cardiac tissue sections showed that MGES alone or co-administered with Ang II markedly increased myocardial SOD1 as shown by the enhanced red fluorescent immunostaining in heart tissue from MGES or Ang II + MGES treated animals (Figure 6). The merged images show the cellular SOD1 immunofluorescence co-localized with the nucleus counterstained with DAPI. Quantification of the immunohistochemical images showed that MGES alone or in combination with Ang II increased the concentration of SOD1 in cardiac tissue sections by approximately 4- and 5-fold, respectively, as compared to heart tissue from Control or Ang II treated animals (Figure 7A).

More importantly, administration of MGES with or without Ang II resulted in a marked increase in cardiac SOD1 activity as compared to heart homogenates from Control or Ang II rats (*p* < 0.01 MGES compared to Control; *p* < 0.001 MGES compared to Ang II; *p* > 0.0001 Ang II + MGES compared to Control or Ang II alone) (Figure 7B). The SOD1 concentration was not significantly different in heart tissue from MGES treated rats as compared to rodents given Ang II + MGES. The observed increase in *Sod1* mRNA in rat cardiac tissue from the MGES or Ang II and MGES groups (Figure 7C) suggests that the enhanced concentration in SOD1 activity was due to transcriptional regulation of *Sod1* mRNA by the extract. Ang II alone had no effect on *Sod1* mRNA. Collectively, these results suggest that MGES upregulates the SOD1, irrespective of hypertension.

### 3.5. Effect of MGES on Cardiac Catalase

**Figure 8:**
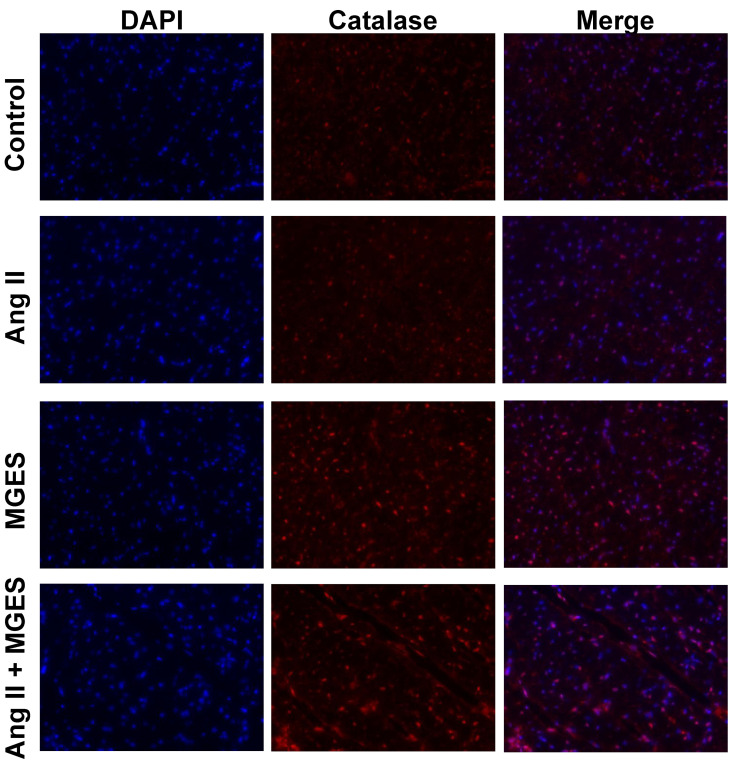
MGES increases catalase immunofluorescence in cardiac tissue. Representative images of cardiac tissue stained with the nuclear counterstain DAPI (blue) and an antibody to SOD1 (red) imaged at ×400 are shown for hearts from rats administered drinking water (Control), Ang II alone (Ang II), MGES alone (MGES), or a combination of MGES and Ang II (Ang II + MGES).

**Figure 9:**
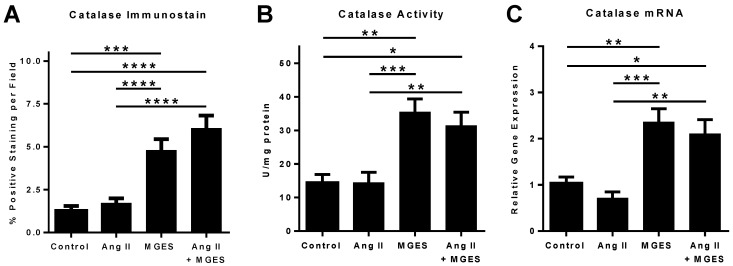
MGES increases catalase protein, activity and mRNA in normotensive and hypertensive rat hearts. Catalase protein, activity or mRNA were measured in heart tissue from rats administered drinking water (Control), MGES alone (MGES), Ang II alone (Ang II) or a combination of MGES and Ang II (Ang II + MGES). (**A**) Quantification of cardiac tissue sections stained with an antibody to to catalase; (**B**) Quantification of catalase activity; and (**C**) Quantification of catalase mRNA. Four representative images per tissue were obtained using the Mantra microscope. Values are mean ± SEM; *n* = 8 per group. The asterisk (*) represents statistically significant comparisons. * *p* < 0.05; ** *p* < 0.01; *** *p* < 0.001; **** *p* < 0.0001.

Similar results were observed for catalase, a key protective enzyme that catalyzes the decomposition of hydrogen peroxide to oxygen and water. MGES with or without Ang II significantly enhanced catalase immunostaining in cardiac tissue sections as compared to Control or Ang II samples as shown visually in Figure 8 by the intensified red fluorescent staining of the MGES or Ang II + MGES tissues as compared to Control or Ang II alone. The merged images show the cellular cardiac catalase immunofluorescence co-localized with the nucleus counterstained with DAPI. Quantification of the immunohistochemical images showed that MGES alone or in combination with Ang II significantly increased the concentration of catalase in cardiac tissue sections, as compared to heart tissue from Control or Ang II treated animals (Figure 9A, *p* < 0.0001 MGES vs. Control; *p* < 0.001 MGES vs. Ang II; *p* < 0.0001 MGES vs. Control or Ang II alone). This suggests that the MGES increased catalase is not related to blood pressure. No difference was observed in catalase protein concentration between the Ang II heart tissue and Control samples as shown by immunohistochemistry (Figure 9A).

MGES or the combination of Ang II and MGES caused a significant increase in cardiac catalase activity as compared to Control or Ang II homogenates (Figure 9B). The catalase concentration was not significantly different in heart tissue from MGES treated rats as compared to rodents given Ang II + MGES. Further, catalase mRNA was increased approximately 2-fold in heart samples from mice treated with MGES or Ang II + MGES as compared to Control or Ang II alone (Figure 9C). Taken together, these results suggest that MGES alters the transcriptional regulation of catalase to increase the concentration and activity of this protective antioxidant enzyme.

## 4. Discussion

In this study, we report that a proprietary muscadine grape extract ameliorated cardiac damage in an Ang II-induced rodent model of hypertension but had no effect on blood pressure. Blood pressure was significantly elevated by Ang II but the MGES did not exacerbate or prevent this increase in blood pressure, suggesting that the extract is safe for hypertensive and normotensive patients. MGES did not block gross cardiac hypertrophy, measured by examining the isolated heart and corroborated by the echocardiographic findings, indicating that MGES had no effect on relative wall thickness. However, co-administration of MGES significantly inhibited the increase in cardiomyocyte mean cross-sectional area compared to Ang II alone, suggesting that MGES administration prevents an increase in the size of cardiomyocytes but that the 4-week treatment may not have been sufficient to cause gross morphometric changes in the size of the heart.

Ang II reduced body weight as previously reported [20,21,22]. Body weight was decreased by Ang II treatment, in the presence or absence of MGES; MGES alone had no effect on body weight or food intake as compared to control rats. Water intake and urine output were also both increased by Ang II treatment, as anticipated, since Ang II is known to increase thirst; however, neither parameter was affected by treatment with MGES. In previous studies demonstrating that Ang II resulted in a similar weight loss, food intake was reduced in rats treated with Ang II [20,21,22]. Conversely, in the current study, the food intake of the rats treated with Ang II with or without MGES was increased. The difference is likely in the time of measurement. In previously reported studies [20,21,22], the food intake was measured during the first week of treatment, while in the current study the food intake was measured at the end of 4 weeks. The continued weight loss with progressive Ang II treatment likely stimulates increased consumption of more food and that may not manifest until a later time point. In addition, previous studies showed that only part of the reduction in body weight was due to a decrease in food intake (in pair-fed rats), with an additional “metabolic” effect accounting for a part of the reduced body weight [20,21,22].

MGES co-treatment prevented an increase in left ventricular filling pressures compared to Ang II-treated animals with no effect on systolic function. Diastolic dysfunction in patients often presents with no early symptoms; if left untreated, maladaptive remodeling including pathological fibrosis associated with diastolic dysfunction will lead to the presentation of symptoms of heart failure. Fibrosis impairs normal ventricular relaxation and diastolic suction and causes an increase in passive stiffness [2]. The pathological response to these changes is increased atrial and ventricular filling pressures for a given volume. Atrial work becomes the main contributor to the effectiveness of ventricular filling. A diagnosis of diastolic dysfunction is typically based on echocardiographic evaluation of left ventricular filling pressures [23]. As LV compliance decreases, E/e′ increases, corresponding to increased left ventricular filling pressures and diastolic dysfunction. MGES inhibited the Ang II-induced increase in E/e′, suggesting improved diastolic function.

Although it is clear that ROS participate in the pathology of cardiovascular diseases, the specific role that reactive species play in the development of hypertensive cardiac damage has not been well characterized. In spontaneously hypertensive rats, oxidative stress precedes the development of hypertension [24]. Clinical data show that markers of oxidative stress (such as MDA and lipid peroxides) are increased in patients with essential hypertension and congestive heart failure [25,26], whereas endogenous antioxidants are significantly decreased in these patients [27]. Despite the evidence of increased oxidative stress in hypertension and hypertension-induced cardiac damage, clinical trials with antioxidants such as Vitamin E and C have not shown promising results [28,29,30]. Explanations for the lack of benefit of natural antioxidants in clinical cardiovascular risk include the type of trial conducted, variances in cohorts of patients, or the theory of a “whole food” being more beneficial than an individual component [31,32,33]. In contrast, prototypical anti-hypertensives (such as ACEIs and ARBs) provide some advantageous effects through reductions in oxidative stress [34,35].

We demonstrate, for the first time, that a muscadine grape extract prevents diastolic dysfunction in association with a reduction in oxidative stress. MGES increased endogenous myocardial SOD1 and catalase protein, activity and mRNA in normotensive and hypertensive rats, suggesting enhanced scavenge of toxic free radicals. MDA and 4-HNE, end-products of lipid peroxidation, increase membrane permeability and alter membrane-bound enzymes/ion channels contributing to impaired cardiac function. Co-administration of MGES prevented upregulation of downstream markers of oxidative stress—MDA, 4-HNE, and urinary 8-OHdG—as observed in patients taking ACEIs or ARBs, suggesting that the MGES and pharmaceutical anti-hypertensives target similar pathways to improve oxidative stress and cardiac function [34,36]. Endogenous antioxidants and antioxidant enzymes are critical in maintaining the redox balance in the myocardium and are required for physiological cellular processes. In patients with ST-segment elevation myocardial infarction, SOD1 is a strong prognostic factor of one-year mortality [37]; rodents with congestive heart failure treated with losartan had increased *Sod* mRNA and activity, suggesting that Ang II signaling plays a role in redox balance [38]. Plasma catalase activity is decreased in patients with various stages of hypertension and treatment with anti-hypertensive drugs reverses this effect [39].

A plethora of studies demonstrate the advantageous effects of polyphenols on cardiovascular health, as documented in several reviews [40,41]. The beneficial role of polyphenols in cardiovascular disease first gained momentum when a clinical study found that individuals who had a higher intake of wine and fat had reduced cardiovascular mortality rates compared to individuals from other countries with a similar amount of fat intake but lower wine consumption [42]. This led to the term “French paradox” which suggests that higher red wine consumption may prevent some of the detrimental effects of increased intake of fat. Since then, polyphenols present in red wine and green tea emerged as potential treatments to improve various cardiac pathologies. One of the most notable polyphenols present in red wine is resveratrol, which has antioxidant and anti-inflammatory properties as well as the ability to reduce blood pressure by upregulating endothelial NO synthase [43]. However, clinical trials using resveratrol in patients with cardiovascular disease were not successful, potentially due to the low bioavailability of resveratrol, the doses used, the length of treatment, and the variability in sample size. Despite studies demonstrating the beneficial role of resveratrol in red wine, a recent global study across 195 countries and territories reported that any amount of alcohol consumption contributes to a loss of health [44], indicating that an alternative way to consume high concentrations of polyphenols in the absence of alcohol would be beneficial. The MGES used in this study is a concentrated form of polyphenols derived from the skins and seeds of muscadine grapes, contains no alcohol, and can be ingested without any deleterious side effects. The major phenolic compounds in this extract include epicatechin, catechin, gallic and ellagic acid and procyanidin, although MGES likely contains other unidentified phenolic compounds given that the total phenolic content exceeded that of the identified compounds. The multitude of phenolic compounds in the preparation likely accounts for the diverse protective actions of the MGES in the myocardium.

The dose of MGE supplementation used in this study (0.2 mg/mL in water or approximately 14 mg/kg per day assuming a consumption of 25 mL of water per day for a 350 g rat) was based upon prior toxicity studies in our laboratory showing that this dose was not toxic in rodents (over a range of 10 to 80 mg/kg per day) and was effective in producing physiological responses in rodents over the same dose range. Using allometric scaling to convert this dose to a human equivalent dose based on body surface area, according to the formulas proposed by Reagan-Shaw et al. [45] and Nair and Jacob [46], an equivalent effective dose in humans would be approximately 2.3 mg/kg per day. We recently completed a Phase I clinical trial, to measure toxicity, using a similar MGES preparation (provided in capsules taken twice daily) in patients with solid tumors, at a dosage range of 320 to 1600 mg/day. For a 60 kg person, this would be equivalent to 5.2 to 27 mg MGES/kg each day, which are higher doses than used for this animal study. The MGES was well-tolerated in patients and a maximal tolerated dose was not reached [14], demonstrating that a dose effective in rats could easily and safely be achieved in humans.

## 5. Conclusions

We show for the first time that a commercially processed muscadine grape extract supplement improves Ang II-induced diastolic dysfunction associated with a reduction in oxidative stress. This MGES was well-tolerated in a Phase I clinical trial with minimal side effects [14]. Although ACEIs, ARBs, calcium channel blockers, β-blockers, and diuretics reduce blood pressure, not all of these therapies preserve diastolic function and myocardial structure; some of these drugs also have detrimental side effects such as intermittent cough and angioedema [4,47]. Because the MGES did not affect blood pressure in either hypertensive or normotensive animals and had cardiac-specific actions, the supplement should not interfere with traditional anti-hypertensive treatment. However, it is important to note that further studies are warranted to determine whether patients will not be at risk for unintended hypotension or drug interactions. Collectively, the results of this study support further clinical assessment on the use of this commercial MGES for the treatment of patients with hypertension or for patients at high risk for developing hypertension, to prevent cardiac damage due to the increase in blood pressure.

## Figures and Tables

**Table 1:** Echocardiographic measurements ^1^.

	Control (A)	Ang II (B)	MGES (C)	Ang II + MGES (D)
EF (%)	76.8 ± 1.2	80.2 ± 0.8	78.3 ± 1.1	76.1 ± 2.1
FS (%)	47.3 ± 1.2	49.8 ± 0.8	48.6 ± 1.1	46.0 ± 1.8
EDD (mm)	8.08 ± 0.12 ^B,D^	6.01 ± 0.22 ^C^	7.61 ± 0.15 ^D^	6.50 ± 0.19
LVPWT (mm)	1.98 ± 0.08 ^B,D^	2.55 ± 0.14 ^C^	2.06 ± 0.06	2.44 ± 0.14
RWT	0.49 ± 0.02 ^B,D^	0.86 ± 0.07 ^C^	0.54 ± 0.02 ^D^	0.76 ± 0.06
IVRT (ms)	20.8 ± 0.3 ^B,D^	32.1 ± 2.6 ^C^	19.7 ± 0.7 ^D^	30.0 ± 2.3

^1^ Echocardiographic analysis of cardiac function and hypertrophy in rats after 4 weeks of treatment with normal drinking water (Control—group A), MGES (group B), Ang II (group C) or the combination of Ang II and MGES (group D). EF: ejection fraction; FS: fractional shortening; EDD: end diastolic diameter; LVPWT: left ventricular posterior wall thickness; RWT: relative wall thickness (calculated as (2 × PWT)/EDD); IVRT: isovolumic relaxation time. Values are mean ± SEM; *n* = 8 per group. Comparisons with a *p* < 0.05 are labeled.

## Data Availability

All data are in the text, table or figures found in the manuscript.
